# Multilayer CVD graphene electrodes using a transfer-free process for the next generation of optically transparent and MRI-compatible neural interfaces

**DOI:** 10.1038/s41378-022-00430-x

**Published:** 2022-09-26

**Authors:** Nasim Bakhshaee Babaroud, Merlin Palmar, Andrada Iulia Velea, Chiara Coletti, Sebastian Weingärtner, Frans Vos, Wouter A. Serdijn, Sten Vollebregt, Vasiliki Giagka

**Affiliations:** 1 Department of Microelectronics, Faculty of Electrical Engineering, Mathematics and Computer Science, Delft University of Technology, Mekelweg 4, Delft, 2628 CD The Netherlands; 2 Technologies for Bioelectronics Group, Department of System Integration and Interconnection Technologies, Fraunhofer Institute for Reliability and Micro-integration IZM, Gustav-Meyer-Allee 25, Berlin, 13355 Germany; 3 Department of Imaging Physics, Faculty of Applied Science, Delft University of Technology, Lorentzweg 1, Delft, 2628 CJ The Netherlands; 4 Erasmus University Medical Center (Erasmus MC), dr. Molewaterplein 40, Rotterdam, 3015 GD The Netherlands

**Keywords:** Engineering, Other nanotechnology

## Abstract

Multimodal platforms combining electrical neural recording and stimulation, optogenetics, optical imaging, and magnetic resonance (MRI) imaging are emerging as a promising platform to enhance the depth of characterization in neuroscientific research. Electrically conductive, optically transparent, and MRI-compatible electrodes can optimally combine all modalities. Graphene as a suitable electrode candidate material can be grown via chemical vapor deposition (CVD) processes and sandwiched between transparent biocompatible polymers. However, due to the high graphene growth temperature (≥ 900 °C) and the presence of polymers, fabrication is commonly based on a manual transfer process of pre-grown graphene sheets, which causes reliability issues. In this paper, we present CVD-based multilayer graphene electrodes fabricated using a wafer-scale transfer-free process for use in optically transparent and MRI-compatible neural interfaces. Our fabricated electrodes feature very low impedances which are comparable to those of noble metal electrodes of the same size and geometry. They also exhibit the highest charge storage capacity (CSC) reported to date among all previously fabricated CVD graphene electrodes. Our graphene electrodes did not reveal any photo-induced artifact during 10-Hz light pulse illumination. Additionally, we show here, for the first time, that CVD graphene electrodes do not cause any image artifact in a 3T MRI scanner. These results demonstrate that multilayer graphene electrodes are excellent candidates for the next generation of neural interfaces and can substitute the standard conventional metal electrodes. Our fabricated graphene electrodes enable multimodal neural recording, electrical and optogenetic stimulation, while allowing for optical imaging, as well as, artifact-free MRI studies.

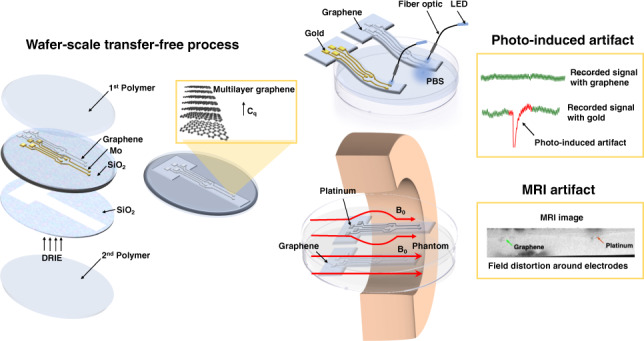

## Introduction

Neural interfaces are tools that enable bidirectional interactions with the human nervous system. To allow for personalized therapies, which is the ultimate goal of bioelectronic medicine, the functional neural behavior has to be well understood. Conventional neural recording and stimulation methods provide insufficient spatio-temporal resolution for neuroscientific research^1^. In addition, it is of paramount importance to monitor neural activity systematically to uncover the interconnections between the neurons and neural clusters. In recent years, several methods such as optical imaging (e.g., calcium or fluorescence imaging)^2,3^, optogenetics^3,4^, and magnetic resonance imaging (MRI)^5,6^ have emerged to assist neuroscientists to decipher the neural structure and function. These, combined with electrical neural recording and stimulation in a multimodal fashion, can pave the way towards a much deeper understanding and mapping of the nervous system^3,4,7^.

Conventional noble metal electrodes, such as gold (Au) or platinum (Pt), are the most common tools for recording neural activity and stimulating neurons due to their good electrical performance, high biocompatibility, and chemical stability. However, due to their opaque nature, they prevent any in vivo optical imaging at the site of stimulation (underneath the electrodes). In addition, due to photoelectrochemical effects, Au electrodes might produce photo-induced artifacts when used for electrophysiology in optogenetic devices^8,9^. Platinum-iridium (Pt-Ir) alloy electrodes, on the other hand, cause image artifacts in MRI due to the magnetic susceptibility of Pt being different from that of the surrounding tissue^10,11^.

Therefore, there is a need for optically transparent and MRI-compatible electrodes. Indium-Tin-Oxide (ITO) and carbon-based electrodes are the most commonly used transparent conductive electrodes. However, ITO cannot be used in flexible devices due to its brittleness that might cause crack formation^12–14^. Among all transparent carbon-based electrodes, graphene is the most attractive material due to its high thermal/electrical conductivity, broad-spectrum transparency, and flexibility^15^. In addition, graphene-coated copper wires^16^ and graphene-fiber electrodes made of graphite oxide^17^ have been proven to be MRI compatible due to their magnetic susceptibilities being close to that of tissue. Therefore, graphene has the potential to be the ideal electrode material candidate for the next generation of optically transparent, and MRI-compatible multimodal neural interfaces.

The majority of research on graphene-related materials concerns graphene-oxide (GO) and reduced-graphene-oxide (rGO) materials. However, due to the electrically insulating properties of GO, its combination with other conducting materials, such as conductive polymers and metals, is necessary to fabricate electrodes. rGO’s large effective surface area leads to low impedance and high charge-injection capacity (CIC) that are both important for neural recording and stimulation^18^. However, its electrical conductivity does not reach that of pristine graphene^19^. More importantly, a cytotoxicity concern towards different types of cells using GO and rGO has been raised recently^20^.

The most common fabrication method for growing graphene is chemical vapor deposition (CVD) which has the advantage of simplicity and the possibility to create high-quality graphene on a metal catalyst that can span a large surface area^21^. However, the required high graphene growth temperature (usually ≥ 900 °C) prevents direct graphene growth on wafers with already present polymers, a fundamental component of flexible implants. Therefore, current state-of-the-art graphene electrode fabrication has been mostly focusing on graphene transfer processes, where graphene is grown on a copper (Cu) catalyst, and subsequently transferred to the required polymer used for the implant^22–25^. Sacrificial polymer supporting layers, such as polymethylmethacrylate (PMMA), facilitate the transfer process. This method, despite its popularity, has reliability and scaling issues^26^ regarding preserving the quality of the material after transfer, polymer residues from the supporting layer, or an additional cleaning process to remove any polymer residues^27^. Finally, metallic particles from the, typically, non-biocompatible Cu catalyst layer can impact the implant’s biocompatibility. Apart from that, in such processes the first polymer layer is present before the graphene transfer. This limits the electrode post-processing options that have the potential to e.g., improve the conductivity^28^.

Other techniques to fabricate graphene electrodes, such as direct laser pyrolysis of porous graphene on a polyimide substrate^29^, or laser carbonization of parylene-C to create graphitic carbon as a coating on metal electrodes^30^, have also been reported. However, to date, laser pyrolysis fabrication has been successfully used only for devices with relatively large electrodes (200–700 μm diameter). The main limitation of this technology is the laser resolution, in comparison with the resolution achieved by photolithographic methods, crucial for miniaturization and the formation of high-density arrays. In addition, these low-quality carbon-based electrodes are not highly optically transparent and suffer from reproducibility issues.

Therefore, using CVD graphene is, so far, the best approach for developing neural electrodes. CVD graphene itself can be created as a monolayer or multilayer, depending on the metal catalyst and the process parameters used^31^. Although monolayer graphene has shown compatibility with neuro-imaging and optogenetics^27^, previously reported works suggest that monolayer graphene in an undoped state suffers from low sheet conductivity^32^. This prevents the use of graphene instead of long metal tracks, reducing the total implant transparency. Moreover, graphene made of fewer layers is more prone to damage during the fabrication and implantation processes.

On the other hand, increasing the number of graphene layers reduces the sheet resistance but also reduces the optical transparency^33,34^. Recent research in the field of supercapacitors showed that multilayering of graphene (up to a threshold of 4 to 6 layers) could result in higher electrochemical capacitance^35^, and previously reported stacked four monolayer graphene neural electrodes have demonstrated good electrochemical characteristics^36^. However, literature suggests that adding graphene layers in a transfer-based process requires more transfer steps, which, in turn, leads to more polymer residues between layers and therefore lower optical transparency^34^.

The aim of the current study is to use CVD multilayer graphene to create fully-transparent and MRI-compatible neural electrodes with better electrochemical performance. To prevent the presence of polymer residues caused by the transfer process, but also, to make the process more compatible with conventional wafer-scale fabrication and post-processing technologies, we have adapted the process reported in^37^, which uses a transfer-free method to grow graphene on a Molybdenum (Mo) catalyst^38^, to create the neural electrodes. This method enables the fabrication of a multilayer graphene electrode without any transfer involved. The electrodes’ impedance, charge storage capacity (CSC), and CIC are assessed and compared to Pt and Au electrodes with the same size and geometry. In addition, the developed electrodes were assessed for compatibility with optogenetic stimulation and MRI, versus Au and Pt electrodes, respectively.

## Methods

### Fabrication process

#### Suspended graphene electrode

**Fig. 1:**
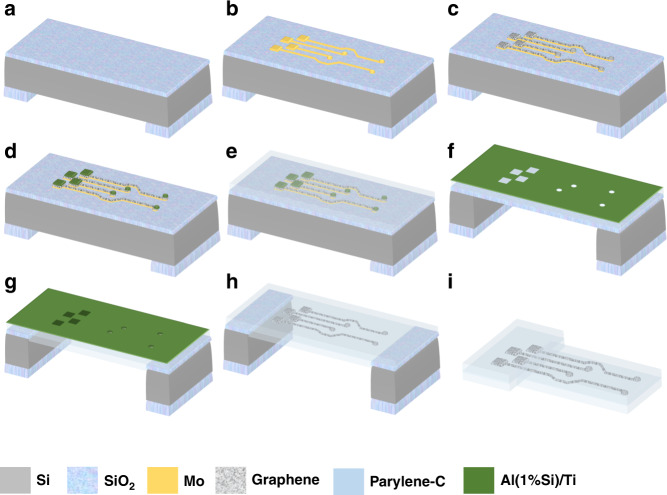
Wafer-scale transfer-free fabrication process steps of graphene-based neural electrodes. Fabrication process steps **a** Oxide deposited on both sides of a DSP Si wafer, patterned, and etched on the backside, **b** Mo deposition and pattern, **c** Graphene growth, **d** Al (1%Si)/Ti deposition and pattern on the electrodes and contact pads, **e** Parylene-C deposition, **f** Al/Ti hard mask deposition and pattern for parylene etching followed by a DRIE process, **g** Frontside oxide removal followed by Mo wet etching, second parylene deposition on the backside, and parylene etching on the frontside, **h** Hard mask wet etching, **i** Cutting the sample.

Multilayer graphene neural electrodes were fabricated as illustrated in Fig. 1.

First, 2 and 4 μm plasma-enhanced chemical vapor deposition (PECVD) oxide was deposited on the front-and backside of a double-sided polished (DSP) 100 mm silicon (Si) wafer (Fig. 1a). The backside oxide is patterned and etched to define the area for a subsequent deep reactive ion etching (DRIE) step. Next, 50 nm molybdenum (Mo) is sputter-deposited at 50 °C on the frontside of the wafer, which serves as the catalyst metal layer for graphene growth. After Mo deposition, lithography steps are employed to define the final design of the electrode array and tracks (Fig. 1b). Etching of the Mo layer is then performed at 25 °C using an ICP etcher with 50 W RF power, 500 W ICP power, 5 mTorr pressure, and 30 and 5 sccm Cl_2_ and O_2_ gas flows, respectively. Graphene is selectively grown on Mo as shown in Fig. 1c using a CVD process (Aixtron Black Magic Pro tool) at temperatures of about 935 °C using 960, 40, and 25 sccm of Ar, H_2_, and CH_4_ gas flows, respectively, at 25 mbar pressure for 20 min and cooled to room temperature under an Ar atmosphere.

The flexible, polymeric-based encapsulation is added in the next step and subsequently the electrodes and contact pads are exposed. A layer of aluminum (Al) is needed to prevent damaging the graphene layer while etching the polymer over the electrodes and contact pads. However, since the adhesion of Al to graphene is poor, an additional titanium (Ti) layer, due to a better microstructure of the film^39^, is needed to act as an adhesion promoter. Hence, prior to polymer deposition, the Al (1%Si)/Ti stack (100 nm of Ti, followed by 675 nm of Al) is sputtered at 50 °C on top of the existing graphene layer and photolithographically patterned (wet etching performed using a 0.55% hydrofluoric acid (HF) solution) to cover the graphene features (Fig. 1d).

Then, 10 μm of parylene-C is CVD deposited at room temperature (using a SCS PDS 2010 parylene coater) (Fig. 1e). Next, in preparation for the upcoming polymer etching step, a hard mask of 500 nm/100 nm Al (1%Si)/Ti is sputter-deposited (at 1 kW, 25 °C) and patterned (dry etched at 25 °C using an ICP etcher with 50 W RF power, 500 W ICP power, 5 mTorr pressure, and 30 and 40 sccm Cl_2_ and HBr gas flows, respectively, with a long over-etching time with 15 and 30 sccm Cl_2_ and HBr gas flows, respectively, to remove potential Al particles from the polymer layer) (Fig. 1f). The hard mask deposition temperature is intentionally kept low to prevent exceeding the parylene glass transition temperature and avoid crack formation.

Finally, a DRIE process on the backside of the wafer lands on the frontside oxide (Fig. 1g), which is then plasma-etched (using an AMS110 etcher (Alcatel) with 300 W RF power, and 17, 150, and 18 sccm C_4_F_8_, He, and CH_4_ gas flows, respectively). Mo is removed at this stage by wet etching in hydrogen peroxide (H_2_O_2_). Graphene will not be accidentally removed in this step as it has already adhered well to the top polymer. Subsequently, the second parylene layer is deposited on both sides of the wafer encapsulating the implant.

To remove the second deposited parylene layer on the frontside and expose the electrodes and contact pads, the frontside parylene is plasma-etched (using the AMS110 etcher (Alcatel) with 40 W LF power, 15 sccm of SF_6_ and 185 sccm of O_2_), landing on the Al protective layer, which is then, together with the hard mask, removed in 0.55% HF (Fig. 1h). At this point, the graphene layer sandwiched between two layers of parylene-C with exposed graphene on the electrodes and contact pads is ready to be cut out of the Si frame (Fig. 1i).

Details of the mask design used for the electrode arrays can be found in Fig. S1.

#### Graphene, Pt and Au electrodes on Si

For rapid prototyping and to investigate and compare the electrode properties of graphene with those of Pt and Au of similar size and geometry, non-suspended devices were fabricated. For this version, parylene insulation is substituted by photoresist to simplify the processing. The fabrication process for these devices is shown in Figs. S2, S3, for graphene and metal (Pt and Au) electrodes, respectively. To contact the electrodes, stainless steel wires are attached to the contact pads using silver (Ag) ink, subsequently covered with a drop of polydimethylsiloxane (PDMS) to enhance the mechanical stability.

For the graphene variant, the devices are at this point placed inside a H_2_O_2_ bath to remove Mo only on the electrodes. The Mo is kept on the contact pads to make a better contact with the attached metal wire.

### Electrode characterization

#### Sheet resistance and optical transmittance

Different growth times (20, 40, and 60 min) were used to create graphene with various thicknesses. Longer growth times result in a larger number of layers. To compare these, both their sheet resistance and optical transmittance are measured. For the sheet resistance, Van der Pauw structures were made, and four-point probe measurements were performed with a Cascade Microtech probe station (see Fig. S4 for more details).

To evaluate the optical transmittance, graphene sheets were grown and transferred to a glass microscope slide (details on the transfer method can be found in Fig. S5). The optical transmittance measurement was conducted using a PerkinElmer Lambda 950 UV/Vis spectrophotometer (PerkinElmer, Waltham, Massachusetts). The wavelength range for the measurement was from 300 to 900 nm. Reference measurements were also performed for only the glass slide. The number of graphene layers can be calculated from the optical transmittance by calculating the total absorbance of the multilayer graphene and comparing it with 2.3% absorbance of a monolayer graphene^40,41^.

To evaluate the quality of a transparent conductive film, a figure of merit (FOM) is used; it is calculated for all graphene thicknesses based on the optical transmittance (T) at 550 nm wavelength and the sheet resistance (R_*sh*_) and can be found in the Supplementary Notes.

#### Electrochemical impedance spectroscopy

Electrochemical impedance spectroscopy (EIS) was used to assess the electrochemical properties of the electrodes. The measurements were performed in phosphate-buffered saline (PBS) in a three-electrode setup with a Pt electrode (3 mm diameter (BASI Inc.)) as a counter electrode (CE), a leakless miniature silver/silver chloride (Ag/AgCl) (eDAQ) as a reference electrode (RE), and the graphene, Au and Pt electrodes fabricated in this work as the working electrodes (WE). The setup was kept inside a Faraday cage during the measurements. All the electrodes were connected to a potentiostat (Autolab PG-STAT302N) that applied a 10 mV RMS sinusoidal voltage between the WE and the RE and measured the current between the WE and the CE^42^. Finally, the impedance magnitude and phase were plotted over frequencies ranging from 1 Hz to 100 kHz.

#### Cyclic voltammetry

Cyclic voltammetry (CV) is frequently used to calculate the amount of charge that an electrode can inject into the tissue^43^. This measurement was also performed using the same three-electrode setup. The water window for graphene was chosen from −0.8 to 0.6 V and used as the CV potential range. As the CSC highly depends on the scan rate, the measurements were performed with various scan rates (0.1, 0.2, 0.6, and 1 V/s). Both the total and cathodic CSC were calculated.

#### Voltage-transient measurements

Voltage-transient measurements are used to estimate the maximum charge that can be injected by means of a constant current stimulation pulse^42–44^. The voltage transient was recorded in the same three-electrode configuration by applying a cathodic-first biphasic symmetric current pulse between the WE and CE (1 ms pulse width, 100 μs interphase delay) in the PBS solution. In the voltage transients between the WE and the RE, an immediate resistive potential drop (access potential (Va)) is observed at the onset of the cathodic pulse followed by a gradual potential decrease due to the capacitive charging of the electrode-tissue interface. The potential reaches its minimum value at the end of this pulse. The interface polarization (Ep) is evaluated by eliminating the resistive potential drop from this minimum potential (Ep = Emin − Va). Next, the applied current amplitude is increased until the interface polarization reaches the cathodic water window extracted from the CV measurement. It should be noted that the anodic interface polarization must also not exceed the anodic water window. Finally, the maximum cathodic CIC of the electrode is calculated based on the maximum current amplitude multiplied by the pulse width and divided by the electrode surface area^44^.

#### Photo-induced artifact test

When shining light on the metal electrode, electrons from the metal surface might be ejected and a small transient potential is created that could interfere with the recorded signal from the neurons. This signal is created due to the photoelectrochemical effect and is called a photo-induced artifact^8,9^.

Here, we tested our multilayer graphene in comparison with gold electrodes using an optical fiber coupled with an 470 nm LED. The setup used for this test is shown in Fig. S6. A safe range of light stimulation intensity for in vivo experiments is up to ~75 mW/mm^2^ for short pulses from 0.5 to 50 ms^45^. In this experiment, rhythmic rectangular pulse stimulation with 10 ms pulse duration at 10 Hz and 50 mW/mm^2^ light intensity was applied to both graphene and the Au electrodes while immersed in a PBS solution. The power spectrum of the recorded signal was investigated for light-induced artifacts. In addition, this test was performed for three different graphene thicknesses to compare the effect of thickness on the produced artifact.

#### MRI compatibility test

To investigate the MRI compatibility of multilayer graphene and Pt electrodes, samples were prepared as follows. To simulate a brain-tissue environment, a phantom was prepared by dissolving 1 g agarose in 100 ml PBS in a Petri dish, where the suspended graphene (Fig. 1) and Pt electrodes were subsequently immersed, and any bubbles were removed using a Q-tip. Finally, the phantom was solidified and placed in a water bath to mitigate the effect of susceptibility artifacts at the edge of the phantom caused by the phantom–air interface to be able to detect potential artifacts from the electrodes.

An image artifact is usually detected as a specific signal dropout that clearly obstructs a portion of the image around the electrodes and prevents visualization of brain structures where neural signals are recorded, or electrical stimulation is applied. The MR images of the phantom were acquired with a clinical 3 T scanner (Philips Ingenia, Best, The Netherlands). The following sequences were used to acquire MRI images: (1) High resolution 3D T2^*^-weighted dual-echo gradient recalled echo (GRE) sequence; (2) Multi-slice GRE sequence with single-shot EPI (echo-planar imaging) readout; (3) Low resolution T2^*^ mapping performed with a multi-echo GRE sequence; (4) Ultra high resolution B_0_ mapping based on multi-echo GRE phase imaging.

B_0_ maps are analyzed to quantitatively assess B_0_ field distortion introduced by the electrodes. A region-of-interest (ROI) is defined to detect the field shifts induced by the electrodes. Then, a background field removal (BFR) method is performed using a high-pass or Gaussian filter with a standard deviation of 23 to remove the field distortions originating from outside of the ROI. The sequences and their corresponding parameters to acquire the MRI images are provided in detail in Table S1.

## Results

### Fabricated devices

**Fig. 2:**
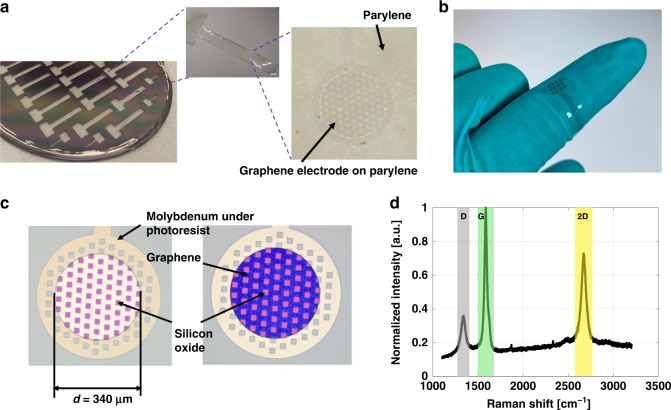
Suspended multilayer graphene electrodes with different polymers (Parylene C and PDMS). **a** Suspended graphene electrodes with parylene-C substrate, **b** Suspended graphene electrode with PDMS substrate, **c** Optical image of the electrode before (yellow) and after (blue) Mo removal, **d** Raman Spectroscopy on graphene electrodes.

The final suspended graphene electrode with parylene substrate is shown in (Fig. 2a). The polymer layer can also be substituted with PDMS based on the application and its required mechanical properties. The suspended graphene electrode with a larger number of electrodes and contact pads with PDMS substrate is shown in (Fig. 2b).

Optical images of the 340 μm diameter electrodes before and after Mo removal are shown in Fig. 2c. The holes on the electrode surface are related to the mask design leaving the device with a surface area of 68,320 μm^2^ as explained in Fig. S1.

Raman spectroscopy using a laser with a 633 nm wavelength on the electrode surface was performed after Mo removal to confirm the presence of graphene on the electrode surface. As shown in Fig. 2d, three distinct peaks can be observed: a D peak (gray) at 1337 cm^−1^ with a full-width at half maximum (FWHM) of 61.02 cm^−1^, a G peak (green) at 1586 cm^−1^ related to the sp^2^ C–C bonds forming the graphene lattice and having a FWHM of 33.32 cm^−1^, and a 2D peak (yellow) around 2670 cm^−1^ with a FWHM of 62.20 cm^−1^. The ratio between the intensities of the D and the G peaks (I_*D*_/I_*G*_ = 0.38) indicates the defects in the graphene layer, which in this case indicates a low number of defects after Mo removal. This ratio matches with the reported values for graphene on Mo for gas sensing applications^46^. The ratio between the intensities of the 2D and the G peaks (I_2*D*_/I_*G*_ = 0.74) confirms the presence of multilayer graphene as the ratio is less than 1^40^. In addition, from the shape of the single-peaked 2D band, it can be postulated that the graphene is turbostratic^47,48^.

### Sheet resistance and optical transmittance

**Fig. 3:**
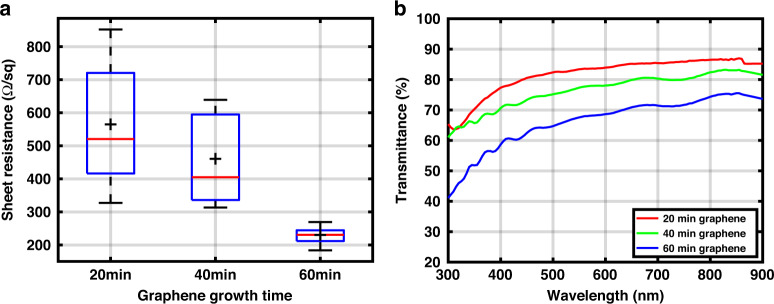
Sheet resistance and optical transmittance characterization of graphene with different thicknesses. **a** Sheet resistance of three different graphene recipes (growth time: 20, 40, and 60 min) showing the maximum, upper quartile, median (red line), average (plus sign), lower quartile, and minimum values, **b** Optical transmittance measurements for different graphene growth times (the effect of the glass slide is removed).

The sheet resistance (R_*sh*_) was measured on 27 Van der Pauw structures for different graphene growth times. The average (plus sign) values for R_*sh*_ are depicted in the box plot in Fig. 3a and reported in the Table 1 for 20, 40, and 60 min graphene growth times. There was a strong correlation between the R_*sh*_ and the location on the wafer for all conditions. The structures in the center of the wafer showed the lowest R_*sh*_, and the structures towards the edge showed higher R_*sh*_. This is possibly due to the single zone heating element in the chamber causing a higher temperature close to the center of the wafer, which results in thicker graphene with a lower defect density.

**Table 1:** Graphene with three different growth times with measured optical transmittance, calculated number of layers, sheet resistance, and FOM.

Growth time	T (%) 550 nm^a^	No. of layers	R_sh_ (Ω/sq)	FOM
20 min	83.5	7	565	3.53
40 min	77.6	10	461	3
60 min	67.5	17	230.5	3.76

^a^These values were calculated for only graphene layers after removing the contribution of the glass slide.

Furthermore, the average R_*sh*_ was lower for a longer growth time. The variation of the R_*sh*_ over the wafer was smaller for the graphene with a longer growth time. That could be explained by the isothermal growth process of graphene, which indicates that with the increased thickness of graphene, the growth rate is slower as carbon has to diffuse through a thicker carbon layer. It has recently been shown for graphene grown on a Nickel (Ni) catalyst that the rate of isothermal graphite growth slows down with increasing exposure time, which might be due to the increased coverage of the catalyst surface with graphite that blocks the precursor supply from the Ni catalyst^49^. Another explanation is the low solubility of Mo (0.0026 weight % at 1000 °C) for carbon atoms. Mo will be saturated faster in the middle, and thus the thickness will not increase further. Therefore, we postulate that with a longer growth time, the thickness of the graphene on the edges of the wafer becomes more similar to the thickness in the center.

Optical transmittance measurements performed on graphene grown for 20, 40, and 60 min after removing the contribution of the glass slide are shown in Fig. 3b. The optical transmittances for graphene at 550 nm are presented in Table 1. The optical transmittance at 550 nm is typically used for the calculation of graphene number of layers^41^. According to these measurements, 20, 40, and 60 min graphene growth times lead to ~7, 10, and 17 graphene layers, respectively. These confirm that increasing the growth time increases the thicknesses of graphene and reduces the optical transmittance.

The calculated FOM is reported in Table 1 for three different growth times. These values are comparable with the result reported for CVD graphene^50^ and also higher than the theoretical value of (2.55) calculated from the same equation for an undoped monolayer graphene in^51^. Finally, a 20 min graphene growth time was chosen for the final electrode to achieve a higher optical transparency.

### Electrochemical impedance spectroscopy

**Fig. 4:**
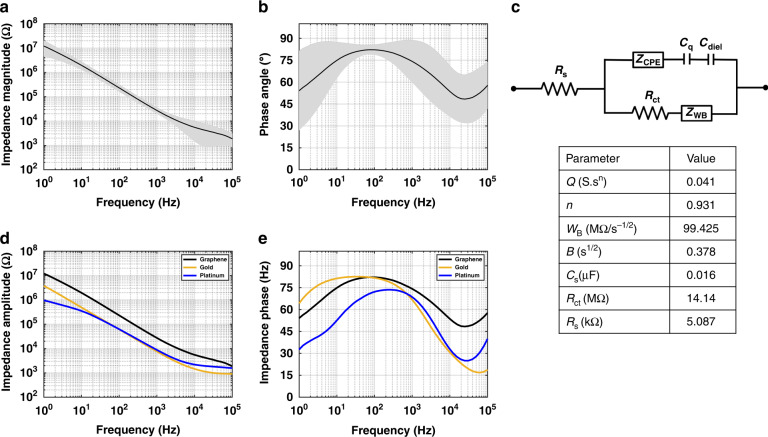
Electrochemical impedance spectroscopy (EIS) for graphene, Pt, and Au electrodes with the same size and geometry. **a** Average impedance magnitude and **b** Phase angle plots (±standard deviation shaded in gray) for fifteen graphene electrodes, **c** Proposed equivalent circuit model for the multilayer graphene electrode and the average values of the parameters used in the equivalent circuit model, **d** Impedance magnitude and **e** Phase angle plots for fifteen graphene electrodes in black (average values), Au electrodes in orange, and Pt electrodes in blue. All electrodes are of the same size and geometry.

EIS measurements were performed on 15 graphene electrodes with 20 min growth time, and the obtained graphs can be found in Fig. 4a, b.

In the Bode plots, the deviation from the average impedance and phase is shown in the shaded gray area. This could be related to slight variations of graphene growth over the Si wafer. EIS measurements were performed for 3 graphene electrodes with 40 min growth time and 3 electrodes with 60 min growth time as shown in Fig. S7. To be able to draw a conclusion a larger number of samples is needed as there are noticeable variations in the impedance at 1 kHz.

The proposed equivalent circuit model for multilayer graphene electrodes is shown in Fig. 4c. In this model, R_*s*_ is the resistance of the solution, Z_*CPE*_ is the constant phase element representing the Helmholtz double layer capacitance. R_*ct*_ is the charge-transfer resistance used to simulate Faradaic reactions and Z_*WB*_ is the bounded Warburg impedance used to simulate the diffusion process.

It was found that the double layer capacitance for graphene is in series with the quantum capacitance (C_*q*_) caused by the limited electronic density of states (DOS)^35,52^. C_*q*_ is relatively small for monolayer graphene and therefore dominant at low frequencies. Recent research shows that, by increasing the number of graphene layers, C_*q*_ is increased and its effect on total capacitance becomes less dominant^35^. It has also been shown that for multilayer graphene another capacitance is added in series with C_*q*_, which is called the dielectric capacitance (C_*diel*_). This capacitance is caused by a shielding effect inside the electrode due to a generated electric field. By increasing the number of graphene layers, this shielding region expands leading to a reduction in C_*diel*_^53^.

The equivalent circuit model was fitted to the Bode plots for all fifteen graphene electrodes (20 min growth time) using the equations presented in the Supplementary Notes. Then, the averages for all parameters were calculated and are presented in the table reported in Fig. 4c. C_*s*_ is the series equivalent capacitance of C_*diel*_ and C_*q*_. n is a constant in the range between 0 to 1 and equals 0.931, which shows the highly capacitive behavior of the constant phase element. Moreover, the high value of R_*ct*_ proves that the electric behavior is mainly capacitive and thus there is little Faradaic current at the electrode-electrolyte interface.

EIS measurements were performed on Au and Pt electrodes with the same dimensions (Fig. 4d, e). The average impedances at 1 kHz, which are typically reported for neural electrodes, are ~7.5, 8.7, and 27.4 kΩ for the Au, Pt, and graphene electrodes, respectively. Furthermore, all electrodes exhibit capacitive behavior at low frequencies. The comparison between the impedance at 1 kHz of the graphene electrodes fabricated in this work and the CVD graphene electrodes fabricated in other works can be found in Table 2. The impedance is normalized to the electrode surface area to ease the comparison.

**Table 2:** Total and cathodic CSC, impedance at 1 kHz, area-normalized impedance, charge-injection capacity, water window of graphene, Pt, and Au electrodes, and a comparison with the state of the art CVD graphene neural electrodes.

	CSC (μC/cm^2^)										
Electrodes		1 V/s	0.6 V/s	0.2 V/s	0.1 V/s	Electrode surface area (μm^2^)	Water window	CIC (μC/cm^2^)	Impedance at 1 kHz (kΩ)	Area-normalized impedance (Ω.cm^2^)	Reference
Graphene (20 min growth time)	Total	972	1298	2425	3549	68,320	−0.8 to 0.6	44	27.4 ± 7.5	18.72 ± 5.1	This work
	Cathodic	631	812	1453	2151						
Platinum (Pt)	Total	940	1131	1611	2012		−0.6 to 0.8	67.33	8.7	5.94	
	Cathodic	726	919	1396	1765						
Gold (Au)	Total	597	757	1272	1663		−0.8 to 0.6	11.7	7.5	5.1	
	Cathodic	454	594	993	1343						
Monolayer graphene (Doped with HNO_3_)	Total				1953	2500	−0.8 to 0.8		541	13.5	^22^
Two stacked Monolayer graphene (Doped with HNO_3_)	Cathodic		22.4 @0.5 V/s			2500	−0.8 to 0.8		908 ± 488	22.7 ± 12.2	^23^
Few layers graphene	Total	910				707	−1.6 to 1.4	150	2650 ± 260	18.73 ± 1.84	^24^
Four stacked monolayer graphene	Cathodic	87.8				31,416	−0.6 to 0.8	57.13	215.7 ± 120.4	67.76 ± 37.8	^25^

### Cyclic voltammetry

**Fig. 5:**
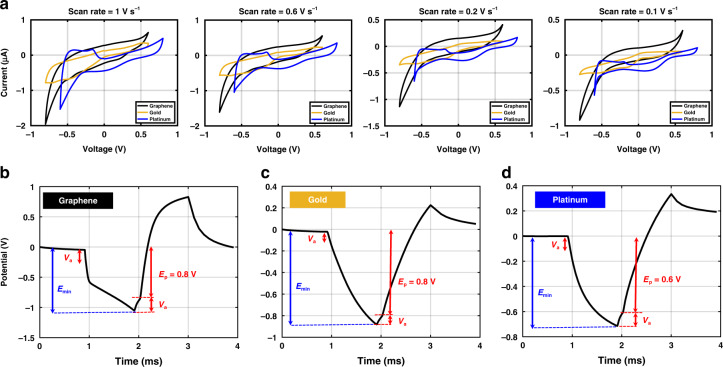
Cyclic voltammetry (CV) and voltage transient measurement for graphene, Pt, and Au electrodes. **a** CV curves for graphene, Pt, and Au electrodes with scan rates 1, 0.6, 0.2, and 0.1 V/s from left to right, respectively, **b** Voltage-transient measurements for graphene, **c** Au, and **d** Pt electrodes.

Cyclic voltammetry was performed on the same 15 graphene electrodes. The CV curves for graphene (20 min growth time), Au, and Pt are shown in Fig. 5a at different scan rates. The CSC values were calculated based on the time integral of the CV curve and are reported in Table 2. The CSC calculated for Au is a lot lower than Pt and graphene. On the other hand, the CSC for graphene is comparable to that of Pt. However, the CSC values for graphene are higher at slower scan rates than those of Pt. This could be related to the high average surface roughness (6.75 nm) measured for 20 min graphene based on atomic force microscopy (AFM) measurements as shown in Fig. S8. At a high scan rate, for electrodes with a high surface roughness, only a fraction of the pores on the electrode surface are accessible for the electrochemical processes. On the other hand, a slower scan rate leads to a slower reactant flux, and therefore, increased accessibility to the electrode surface^54^.

The comparison between the CSC calculated for graphene electrodes with different thicknesses was inconclusive as the variation between the CSC of the electrodes is insignificant. Therefore, a larger number of samples is needed for 40 and 60 min graphene growth to be able to study the impact of thickness on the CSC.

Furthermore, as shown in Table 2, the CSC of our graphene electrodes is 1.8, 36, and 7 times higher compared with the graphene electrodes made from doped monolayer, two stacked monolayer, and four stacked monolayers, respectively^22,23,25^. Moreover, the graphene electrodes reported in^24^ show a high CSC at 1.0 V/s scan rate but still lower than the one reported in this work. Such a high CSC for the graphene electrode reported in^24^ was related to the larger potential window used for the CV measurement.

### Voltage-transient measurements

The results of voltage-transient measurements for graphene, Au, and Pt are shown in Fig. 5b–d. The maximum current amplitude that could be applied to the electrodes before exceeding the safe potential window are 30, 8, and 46 μA, for graphene, Au, and Pt, respectively. The calculated CICs are 44, 11.7, and 67.33 μC/cm^2^ for graphene, Au, and Pt electrodes, respectively. It should be emphasized that by reducing the current pulse width, the current amplitude could be increased to ensure that the current is high enough to elicit neural activation, as pulse widths shorter than 0.6 ms are generally employed in neural stimulation^43^. However, this result still can be used as an indication of the CIC for neural stimulation.

### Photo-induced artifact test

**Fig. 6:**
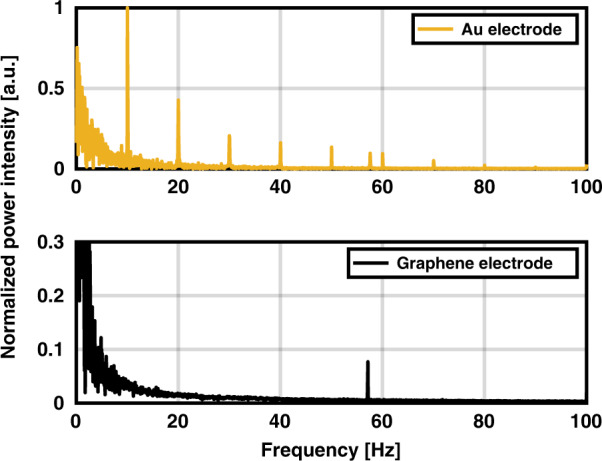
Photo-induced artifact test for graphene and Au electrodes in a PBS solution. Normalized power spectrum of the recorded signal from Au and graphene electrodes (zoomed-in) after shining light with 10 Hz frequency.

The power spectra of the recorded signals for Au and graphene electrodes while shining 10 Hz light pulses on their surface are shown in Fig. 6. The spectra are normalized to the first harmonic of Au electrode. No artifact was detected in the power spectrum of graphene electrodes. On the other hand, for Au electrodes, the fundamental frequency component, but also harmonic components at 20, 30, 40, 50 Hz, etc., are observed. The measurement was repeated for graphene grown with different thicknesses and no artifacts were revealed.

### MRI compatibility test

**Fig. 7:**
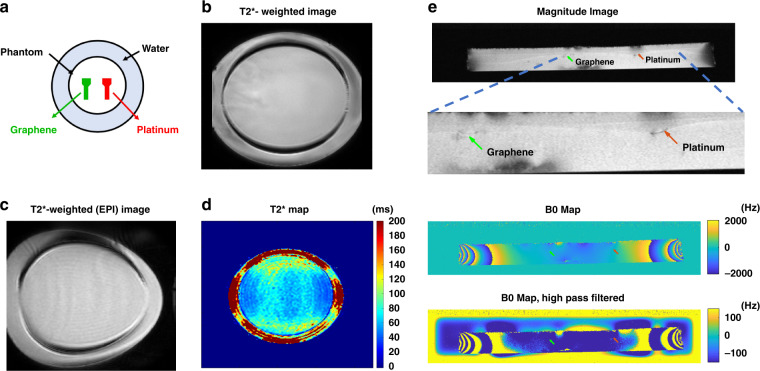
MRI compatibility test for graphene and Pt electrodes immeresed in a phantom. **a** Immersed Pt and graphene electrodes in a phantom, **b** T2^*^-weighted image with no artifact from the electrodes, **c** T2^*^-weighted image acquired with EPI readout resulting in an artifact-free imaging, **d** T2^*^ maps of the electrodes without any artifact, **e** Baseline magnitude image, B_0_ maps, and the high-pass filtered image of the B_0_ maps of the electrodes.

As shown in Fig. 7a, an MRI compatibility test was performed for graphene and Pt electrodes in a 3 T MRI scanner. In this test, the eventual introduction of susceptibility artifacts that would then lead to signal dropout was investigated. To do this, T2^*^-weighted images were acquired because they accentuate local susceptibility effects. However, no electrode-related image artifact was detected in these images (Fig. 7b). Therefore, EPI images, which are even more sensitive to B_0_ inhomogeneity and then actual T2^*^ maps were acquired. No image artifact was detected around the electrodes in the T2^*^-weighted image shown in Fig. 7c as well. The T2^*^ maps represented in Fig. 7d also did not reveal any artifact around the electrodes. The lack of any artifact around the Pt electrode could be related to the very small thickness (100 nm) of the Pt electrodes.

However, the B_0_ map acquired at a high resolution in a sagittal view shows a differential field response around Pt and graphene electrodes (Fig. 7e). The field shifts induced by the electrodes are much smaller than the spatial inhomogeneity of the main magnetic field. Therefore, the field distortions originating from outside the ROI need to be removed using BFR. Then, the field distortion introduced by the electrodes becomes vaguely visible, and it is apparent that the effect is much stronger for Pt than for graphene. The mean value of the field distortion around Pt and graphene was averaged over fifteen repetitions resulting in 63.33 ± 67.02 and 3.4 ± 5.42 Hz variations around the main magnetic field value (B_0_ = 3 T), respectively. This shows that the Pt electrode causes about 18.6 times higher magnetic field distortion than the graphene electrode due to its higher magnetic susceptibility than the surrounding tissue.

## Discussion

Multilayer graphene electrodes were fabricated using a wafer-scale transfer-free process. The use of CVD processes for graphene synthesis gives the opportunity of developing graphene layers only over desired areas, since the catalyst used can be patterned before graphene growth. Mo is chosen as a catalyst layer due to the possibility of growing thin and uniform layers of graphene because of its extremely low carbon solubility, thus creating a self-limiting growth process^55^. Moreover, the thermal expansion coefficient (CTE) of Mo in comparison with Cu and Ni is much closer to the one of Si, hence, Mo is less prone to wrinkle creation during high temperature graphene growth^56,57^. Additionally, catalyst residues are an important concern in an implantable device. Cu has shown toxicity after histopathological evaluation in the cerebral cortex and categorized as a toxic material for the human body^58,59^. Mo has shown biocompatibility^60^and biodegradability^61,62^ and therefore, is a great substitute for Cu as a catalyst material for biomedical applications. In addition, energy dispersive X-ray (EDX) analysis performed on our graphene electrode after Mo removal revealed only 0.03% weight percentage of Mo residue on the electrode surface as shown in Fig. S9.

The use of a transfer-free process adds significant advantages to the fabrication process. The graphene transfer method is a complicated process and the graphene layer is prone to crack formation, polymer contamination, catalyst residues, wrinkling, and folding^26^. Therefore, the resulting graphene implant performance might have a variation from device-to-device and wafer-to-wafer.

However, due to the transfer-free process used in this work, less defects and misalignment are expected in a graphene layer compared to transferred graphene. Subsequently, the absence of any polymer residues results in high optical transparency. More importantly, the transfer-free process is more compatible with conventional wafer-scale fabrication processes and results in a higher yield, as shown by the authors in ref. ^63^. This could provide the possibility of monolithic integration of active circuitry to the device prior to graphene growth. The proposed fabrication process can be also an advantageous method for the fabrication of optoelectronic devices.

The process also allows for the addition of arbitrary polymers at the end of the fabrication process based on their mechanical characteristics and the application requirements. Reference^64^ and^65^ also shows the use of a multilayer stack for the encapsulation. In these cases, the mechanical properties of the device can be tuned by changing the thickness of each layer based on the application.

Multilayer graphene could cause a lower sheet resistance for graphene tracks compared to monolayer graphene as the sheet resistance is inversely proportional to the thickness of the film. Moreover, having multiple graphene layers provides additional transport paths for the charge carriers, which increases the conductivity of graphene. Recent research shows that increasing the number of layers to reduce the sheet resistance in a transfer process leads to optical transmittance reduction not only due to the added layers but also due to the polymer residues on each layer from the transfer process. Furthermore, since the transfer process can induce defects in the graphene lattice, for the same number of layers, fewer transfers show lower sheet resistance^33^.

The results obtained by the sheet resistance and optical transmittance measurements in this work show sheet resistance and optical transparency reductions by increasing the graphene growth time. Besides, the additional layers are expected to enhance the mechanical and electrical reliability^66^. Therefore, 20 min graphene growth was chosen to make graphene-based devices that are optically transparent enough to be used for modern neuroscientific research such as optogenetics and in vivo optical imaging. It should be noted that doping could decrease the sheet resistance even further but this was not the focus of this work.

A thorough characterization of the properties of the graphene electrodes presented here was conducted and results are summarized in Table 2.

A comparison between our multilayer graphene with Au and Pt electrodes showed only 3–4 times higher impedance (1 kHz) for graphene electrodes. The multilayer graphene electrodes fabricated in this work showed a lower area-normalized impedance compared to other undoped CVD-based graphene electrodes.

CV measurements showed that our graphene electrodes are comparable to Pt electrodes in terms of CSC. Graphene electrodes outperform Pt electrodes when using slower scan rates for CV measurements. This could be related to the high graphene surface roughness that could be more accessible for ion fluxes at lower frequencies. The CSC at different scan rates was measured to be able to compare the result with state-of-the-art graphene electrodes. It was shown that our multilayer graphene has the highest CSC reported so far for CVD graphene electrodes.

The significant improvement in CSC for the multilayer graphene compared to monolayer graphene could be explained by the effect of the quantum capacitance in series with the double layer capacitance. By increasing the number of graphene layers, the quantum capacitance is increased. Therefore, this capacitance is no longer dominant for multilayer graphene and the total capacitance will be increased.

On the other hand, voltage-transient measurements showed comparable CIC for both graphene and Pt. However, to substitute conventional metal electrodes, the CIC could be further improved using chemical dopants or surface functionalization methods to give graphene the possibility to compete with Pt electrodes. In fact, other transparent materials such as poly (3,4-ethylenedioxythiophene) polystyrene sulfonate (PEDOT:PSS) and carbon nanotube (CNT) with great CIC (up to 15 and 1.6 mC/cm^2^, respectively) and low impedance due to their high surface area are other electrode candidates^43^. These have been added as coating materials on graphene to improve its characteristics^67,68^.

The graphene electrodes in^24^ appear to be capable of higher CIC than what we achieved. This is probably related to the unusually large potential window used in the CV measurement in^24^. A detailed study on the safe potential limit used for CV measurement for graphene material is hence necessary to further appreciate the capabilities of graphene as a stimulation electrode.

Regarding photo-induced artifacts, a previous report for a monolayer graphene electrode tested with a 470 nm light emitting diode (LED) light source did not show any artifact^27^. However, a photo-induced artifact was observed with stacked four monolayer graphene tested using blue laser diodes^25^. Therefore, it was uncertain whether the artifact was induced due to a larger thickness of graphene or due to the different light sources used for this test.

The photo-induced artifact test performed in this work using an LED light source, showed no artifact on the power spectrum of the recorded signal picked up from the graphene electrode. However, visible peaks were observed using the Au electrode. The same measurement with different thicknesses of graphene still did not show any artifact. This could prove the lack of dependence of photoelectrochemical effect on the graphene thickness. However, to be able to conclusively argue about such independence, additional characterization would be needed. More importantly, LEDs were used as the light source in this test. It is possible that when a coherent light source, i.e., a laser diode, is used instead, photo-induced artifacts will be generated^9^.

Moreover, it should be noted that for a thorough investigation of the photo-induced artifact, this test must be performed in an in vivo condition as the light scattering and absorption in tissue differs from that in a simple PBS environment. However, this PBS test is a good first indicator and can additionally provide information about the effect of increased thickness on any generated artifact.

The MRI compatibility of graphene encapsulated Cu wires^16^ and graphene fibers^17^ has been recently confirmed. The MRI test performed in this work shows that CVD graphene electrodes encapsulated with parylene-C can be considered MRI compatible. This could be due to the small difference between the magnetic susceptibility of graphene and the human body. The exact value of magnetic susceptibility of graphene is unknown. However, carbon (C) in graphite form is reported to have a highly anisotropic diamagnetic susceptibility (−8.5 ppm)^69^, which is very close to that of brain tissue (−9.2 to −8.8 ppm)^70^.

On the other hand, Pt electrodes were expected to show image artifact in MRI. However no artifact was detected. Therefore, using Pt electrodes with a larger thickness or in an MRI scanner with a higher magnetic field strength (7 T or more) might generate even higher magnetic field distortion leading to more image artifacts.

No substantial heating was detected with a room temperature IR thermometer. However, the use of a phantom instead of real tissue might lead to a different temperature distribution and thus a different degree of image artifacts. Therefore, an in vivo MRI test with graphene electrodes implanted would be advantageous.

Apart from a magnetic susceptibility difference, the material conductivity and the eddy currents induced in the material by gradient switching and the RF field might cause MRI artifacts. However, the eddy current induced artifact was assumed to be negligible.

## Conclusion

We presented the development and characterization of fully-transparent CVD-based multilayer graphene electrodes using a wafer-scale transfer-free process for the next generation of optically transparent and MRI-compatible neural interfaces. The electrodes were fabricated directly on a patterned Mo catalyst resulting in a multilayer graphene electrode.

The electrode showed low impedance (27.4 kΩ) at 1 kHz that is quite comparable to those of Au and Pt electrodes with the same size and geometry. A 3.5 mC/cm^2^ CSC was achieved based on CV measurements for graphene at a 100 mV/s scan rate that is the highest value reported for CVD graphene electrodes to date. The CIC was also calculated for graphene electrodes (44 μC/cm^2^) using voltage-transient measurements. Our graphene electrodes illuminated with light pulses with a repetition rate of 10 Hz did not reveal any photo-inducted artifacts for all thicknesses measured. Moreover, the fully-transparent electrodes did not show any image artifact in a 3 T MRI scanner. These results show that graphene multilayer electrodes with a high CSC and a low impedance could be used for the next generation of neural interfaces, enable multimodal electrical and optical recording and stimulation, and substitute the current standard metal electrodes, to additionally allow for MRI studies of the nervous system.

## Supplementary information

Supplementary material
