# Supplementary material for: Multilayer CVD graphene electrodes using a transfer-free process for the next generation of optically transparent and MRI-compatible neural interfaces
Source: Microsyst Nanoeng. 2022 Sep 26;8:107. doi: 10.1038/s41378-022-00430-x (PMC9512798; doi:10.1038/s41378-022-00430-x)
Supplement: Supplementary file 1 — Supplementary material [file 41378_2022_430_MOESM1_ESM.pdf]

Supplementary Notes

Nasim Bakhshaee Babaroud[1]*, Merlin Palmar[1], Andrada Iulia Velea[1,2], Chiara Coletti[3], Sebastian Weingärtner[3], Frans Vos[3], Wouter Serdijn[1,4], Sten Vollebregt[1] and Vasiliki Giagka[1,2]

[1*]Department of Microelectronics, Faculty of Electrical Engineering, Mathematics and Computer Science, Delft University of Technology, Mekelweg 4, Delft, 2628 CD, The Netherlands.

[2]Technologies for Bioelectronics Group, Department of System Integration and Interconnection Technologies, Fraunhofer Institute for Reliability and Micro-integration IZM, Gustav-Meyer-Allee 25, Berlin, 13355, Germany.

[3]Department of Imaging Physics, Faculty of Applied Science, Delft University of Technology, Lorentzweg 1, Delft, 2628 CJ, The Netherlands.

[4]Erasmus University Medical Center (Erasmus MC), dr. Molewaterplein 40, Rotterdam, 3015 GD, The Netherlands

*Corresponding author(s). E-mail(s): n.bakhshaee@tudelft.nl; v.giagka@tudelft.nl.

Contributing authors:

palmar.merlin@gmail.com; A.Velea-1@tudelft.nl; C.Coletti@tudelft.nl; S.Weingartner@tudelft.nl; F.M.Vos@tudelft.nl; W.A.Serdijn@tudelft.nl; S.Vollebregt@tudelft.nl.

Mask design

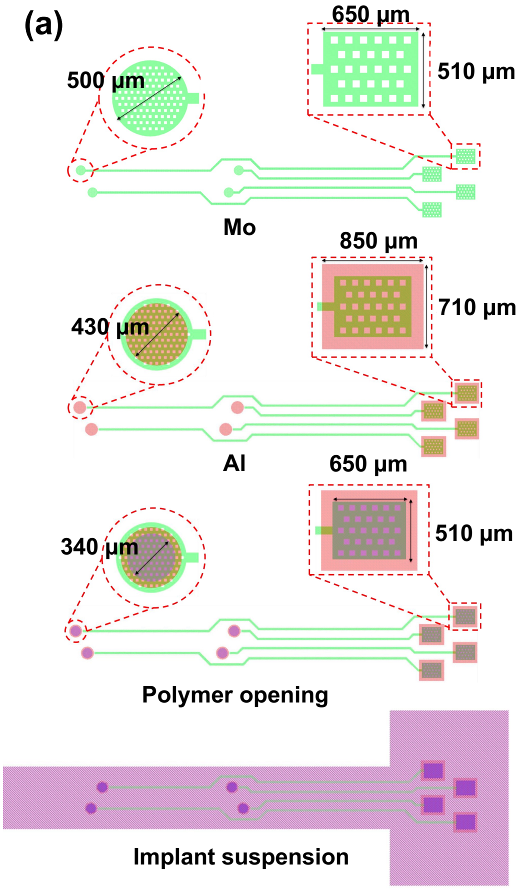

Fig. S1. Masks used for the microfabrication of full implant devices. The electrode diameter is 340 µm which leads to an area of 90792 μm^2. However, the total surface area after subtraction of the holes is 68320 μm^2.

In the Mo/graphene mask, holes are designed to promote a better adhesion of the metal layer (Al (1%Si) / Ti) that will be applied on top of graphene with the layer underneath (SiO_x).

In the Al/metal mask, the metal layer on top of the electrode is smaller such that it can easily be removed from graphene when needed. Moreover, once the metal is being removed, the graphene electrode area should not be fully exposed but protected against possible delamination, at its outermost ring, by the polymer on top (smaller opening on in the polymer opening mask for the electrodes). The metal layer on the contact pads of graphene is slightly larger than graphene contact pads. The reason is the adhesion needed to keep the metal layer on top of graphene until the final steps of microfabrication process.

Additionally, in the polymer opening mask, the dimensions of the openings for the contact pads are the same as for the graphene pads, to ensure that graphene pads are completely covered by the metal layer. This Al metal layer on the contact pad was also designed for soldering wires to the contact pads. However, the openings for the electrodes are smaller than the metal to avoid damaging the graphene layer in case of misalignment of the mask during the lithography steps.

Graphene, Pt and Au electrodes on Si

Graphene electrodes on Si

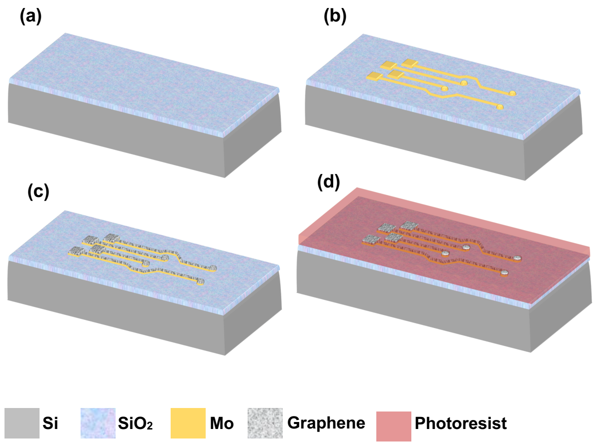

Fig. S2. Fabrication process steps for graphene electrodes on Si. (a) 300 nm wet thermal oxide grown on the frontside of a single-sided polished (SSP) Si wafer, (b) Mo deposition and pattern, (c) Graphene growth, (d) 4μm photoresist coating as an insulation layer. The photoresist is finally patterned and etched only on the electrodes and contact pads.

Pt and Au electrodes on Si

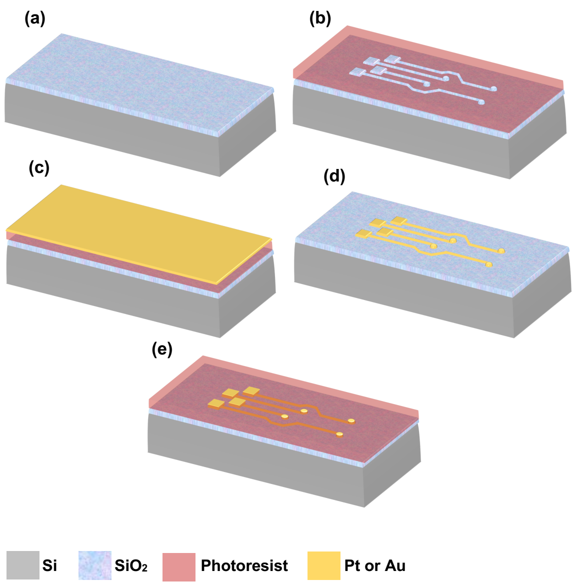

Fig. S3. Fabrication process steps for Pt and Au electrodes on Si. (a) 300 nm wet thermal oxide deposited on the front side of a SSP Si wafer, (b) Photoresist spin-coating and pattern, (c) 100 nm thick Pt or Au e-beam vapor-deposited on a 10 nm Ti adhesion promoter (d) Lift-off process and removing the remaining photoresist layer in an ultrasonic bath (e) Photoresist coating and pattern

Sheet resistance measurement

Van der Pauw structures for four-point probe measurements

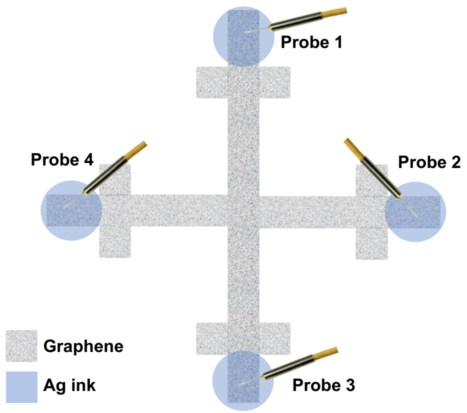

Fig. S4. Van der Pauw structures used for measuring the sheet resistance of graphene layers with different growth times (20, 40, and 60 min). Contact pads were covered with a drop of silver (Ag) ink to prevent any damage caused by the measurement probes on the graphene.

Four-point probe measurements were performed with a Cascade Microtech probe station. The four probes were positioned on the Ag ink dots on the extremities of the Van der Pauw structures. The current was forced between Probes 1 and 2 ($I_{1,2}$), by setting the Probe 1 potential from -1 V to 1 V with 8 mV steps and Probe 2 acting as ground. The voltage was measured between Probes 3 and 4 ($V_{3,4}$). The sheet resistance (R_{sh}) was calculated using the equation below for each point in Ω/sq unit, which were then averaged, excluding the values around I = 0.

$$R_{sh} = \frac{V_{3,4}}{I_{1,2}} \frac{\pi}{ln2}$$

Graphene transfer process

The method used for graphene transfer started with a full wafer with graphene grown on non-patterned Mo. The wafer was then manually diced into roughly 2 cm^2 pieces. These were then placed in a beaker where H_2O_2 was added just until the height of the Si piece with graphene. Mo then started to etch from the sides towards the centre, as illustrated in Fig. S5 (a). After all the Mo was etched, the graphene was separated from the silicon piece and floated on the surface of the liquid while the silicon fell to the bottom of the beaker. DI water was then added very gently with a pipette until the level of the liquid rose about 2 cm. The floating graphene was then scooped on the glass slide. One drop of Triton X100 was added to 1 litre of DI water and 50 ml of triton-water solution was added to the H_2O_2 in the beaker to reduce surface tension. The resulting glass slides with transferred graphene are shown in Fig. S5 (b).

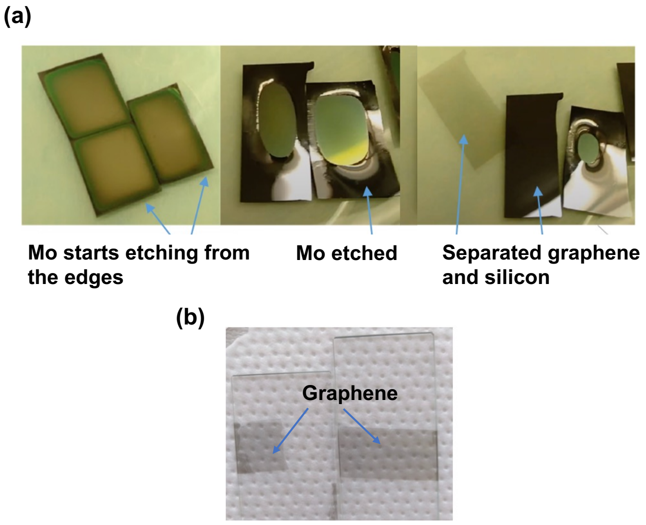

Fig. S5. Sample preparation for optical transmittance measurement, (a) graphene transfer process to glass slides, (b) graphene with 20 min growth time transferred to a glass slide

Figure of merit (FOM)

To evaluate the quality of a transparent conductive film (TCF), the figure of merit (FOM) is calculated based on optical transmittance (T) at 550 nm wavelength and sheet resistance (R_{sh}). Films with low R_{sh} at high optical transmittance show higher performance as a TCF, therefore, a higher FOM corresponds with better TCF performance. The most widely used FOM is defined as follows:

$$FOM = \frac{188.5\,\sqrt{T}}{R_{sh}(1-\sqrt{T})}$$

The FOM calculated based on this equation can be used to evaluate all films with different thicknesses, synthesised from different methods and materials.

The reported values in this work are comparable with the result from graphene electrodes manufactured using a CVD process and also higher than the theoretical value (2.55) calculated by the same equation for an undoped monolayer graphene. However, the calculated FOM is as expected a lot lower than the one of ITO (60 for 40 nm).

Photo-induced artifact

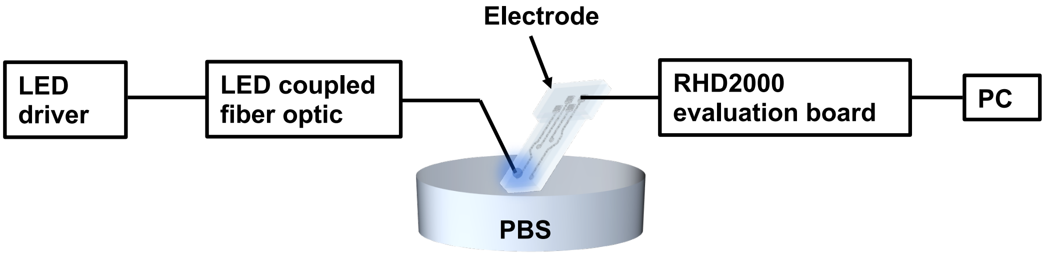

Fig. S6. Setup used for photo-induced artifact test. An Intan RHD2000 Evaluation System was used for data acquisition. A 200 µm diameter fiber was used to shine the light from an LED (M470F1, Thorlabs) on an electrode site. The electrode and the RHD headstage were both put inside a Faraday cage to prevent any interference.

MRI sequences and parameters

Table S1. MRI sequences and parameters used to acquire each image

Sequence	Acquired images	Acquired resolution (mm^3)	Field of view (FOV) (mm^2)	Nr. slices	Nr. echos	T_R (Repetition time) (ms)	T_E (Echo time) (ms)	Flip angle (°)	Sense
Dual-echo GRE	T2*-weighted	0.7×0.7×0.7	150×131	70	2	22	T_{E1} = 6.2, T_{E2} = 15.2	15	-
SSh GRE EPI	T2*-weighted	1.4×1.4×1	150×150	30	1	3681	50	90	-
Multi-echo GRE	T2*-weighted, T2* maps	1.8×1.8×2	200×200	10	15	19	T_{E1} = 1.96, ΔT_E = 1.1	25	Factor = 2
Spoiled GRE	T2*-weighted, B_0 maps	0.2×0.2×7	110×28	1	2	2400	T_{E1} = 15, T_{E2} = 25	70	-

Electrical impedance spectroscopy (EIS)

The EIS data for graphene electrodes using 40- and 60-min graphene growth recipes are shown in the Fig. S6 (a, b), respectively. The impedance of three 40-min graphene electrodes at 1kHz

are 13.1, 47.4, and 56.4 kΩ and the impedance of three 60-min graphene electrodes are 18.2, 23.8, and 47.8 kΩ. Therefore, to be able to draw a conclusion about the influence of graphene number of layers on impedance larger number of samples is required as there are noticeable variations in the impedance for different samples.

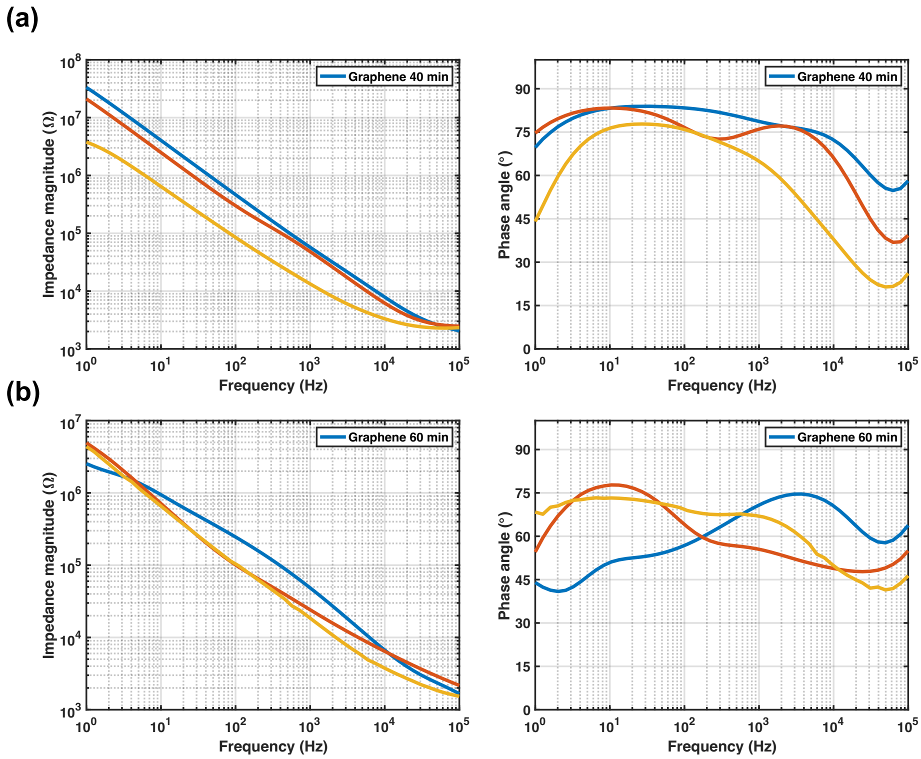

Fig. S7. Average impedance magnitude and phase angle plots (with standard deviation shaded in grey) for (a) three graphene electrodes using 40 min graphene growth, and (b) three graphene electrodes using 60 min graphene growth.

Equivalent circuit model

The equation used for Z_{CPE} in the equivalent circuit model is:

$$Z_{CPE} = \frac{1}{Q(j\omega)^n}$$

where j is the unit imaginary number for which it holds that $j^2 = -1$, ω is the angular frequency being 2π times the frequency of the AC signal (ω= 2πf), Q is a measure of the magnitude of

Z_{CPE}, and n is a constant in the range between 0 to 1. When n = 1, Z_{CPE} is a purely capacitive impedance element, and Q is capacitance; when n = 0, Z_{CPE} is a purely resistive element, and Q is its conductance, the reciprocal of its resistance. For practical electrode–electrolyte interfaces, the Z_{CPE} is used instead of a pure capacitance, accounting for the non-ideal capacitive behavior of the electrochemical double layer.

The equation used for Z_{WB} in the equivalent circuit model is:

$$\boldsymbol{Z_{WB}} = \frac{\boldsymbol{W_B}}{\sqrt{\boldsymbol{j\omega}}} \times \textbf{tanh}\left(\frac{\sqrt{\boldsymbol{j\omega}}}{\boldsymbol{B}}\right)$$

Where W_B is the finite-length Warburg coefficient and B is:

$$\boldsymbol{B} = \frac{\sqrt{\boldsymbol{D}}}{\delta}$$

Where D is the diffusion coefficient and δ is the thickness of the diffusion layer.

Cyclic voltammetry (CV)

The CV scan started at 0 V, then the potential increased until the upper limit. Next, the potential decreased to the lower limit and after that returned back to 0 V. The scan was repeated 3 times to stabilise the signal, and the third scan was used in the calculation of the CSC. The CSC is calculated based on the time integral of the CV curve. The calculated charge was then divided by the electrode surface area (68320μm^2) to obtain the charge density. Finally, the CSC was expressed in $\mu C/cm^2$.

Atomic force microscopy (AFM)

Since any surface roughness greatly increases the CSC due to an increase in the electrochemical surface area of the electrode, the prepared graphene samples were characterized by atomic force microscopy (AFM). AFM was conducted using an NTEGRA Spectra system at ambient conditions. The morphology was captured in the tapping mode with a NSG01 probe. The surface roughness was measured across the 50 × 50 μm^2 areas, calculated as the root-mean square of the height distributions, and then averaged. The phase lag of the AFM probes was measured simultaneously with the topography to achieve better contrast of small topographic features. High-quality images were processed in the standard way using Gwyddion, applying polynomial correction of the background. Then, surface characterization was used to determine the surface roughness.

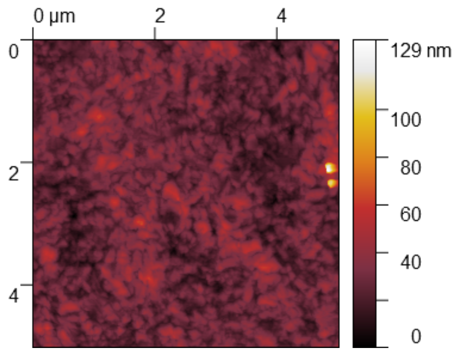

Fig. S8. AFM image of the graphene electrode surface

EDX measurement

For this measurement, Mo is deposited on a Si wafer with a pre-deposited SiO_2 layer. Next, graphene is grown on Mo catalyst. Then, the Mo is etched using a simple wet etching process (using H_2O_2) as was also used in the paper. To verify that there is no Mo residue energy dispersive X-ray (EDX) (FEI XL30 SFEG, w. EDAX Octane Plus detector) analysis was performed. During the EDX measurement the electrode surface after Mo removal was focused using 15 kV electron beam and a spot size of 6. The elemental mapping results acquired after 92 minutes represented in Fig. S9 show the presence of Si, O, C, and Mo and the corresponding spectrum. The indicated areas with graphene and without graphene in the SEM image matches with the C map.

Details of the atomic and weight percent of each element are listed in the inset Table represented on the right side of the diagram.

At the location where normally the Mo peak is found (2.293eV) no clear feature can be distinguished above the noise floor. Upon manually selecting Mo as element, 0.03 weight and 0.01 atomic percentages are attributed to this element. However, the thin layer of graphene is still present and EDX was able to distinguish it.

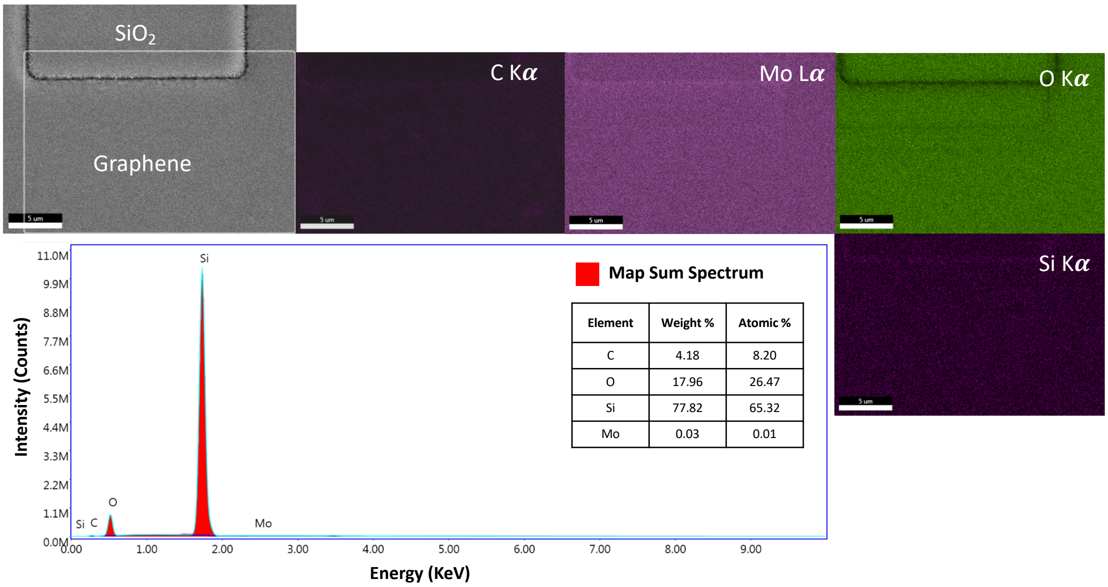

Fig. S9. SEM image, EDX elemental mapping, and the map spectrum of the graphene electrode after Mo removal
